# Quantification of porosity in composite resins delivered by injectable syringes using X-ray microtomography

**DOI:** 10.1080/26415275.2020.1784013

**Published:** 2020-07-06

**Authors:** Bo Wold Nilsen, Mathieu Mouhat, Asbjørn Jokstad

**Affiliations:** Department of Clinical Dentistry, UiT – The Arctic University of Norway, Tromsø, Norway

**Keywords:** Composite resins, porosity, X-ray microtomography

## Abstract

**Objective:**

To assess whether composite polymer resin delivered in compules include pores and the possible effect on the amount of porosity in dental restorations.

**Method and materials:**

Original compules containing unpolymerised composite polymer resin (CPR) were scanned in a micro-CT. Four products were examined, which comprised universal composites (Herculite XRV Ultra, Ceram.X Universal, Tetric Evo Ceram) and a flowable bulk-fill composite (SDR) (*n* = 10 per group). The pore size distribution and amount of porosity (vol.%) were estimated for the unpolymerized and polymerized material used to restore a standardised cavity in a typodont tooth. Manufacturers’ instructions were followed regarding material handling, and polymerisation by use of a calibrated light-curing unit. The pore characteristics and their size distribution, and the amount of porosity in the dental restoration were contrasted with the values measured in the compule. Non-parametric tests were used to analyse differences between the four products.

**Results:**

All the composite polymer resin compules contained unpolymerised material that included pores. The universal composite compules included pores predominantly in the sub-100 µm sizes. In contrast, the flowable bulk-fill compules included a few pores with a diameter >100 µm, which were assumed to be air-bubbles. The unpolymerised material within the compule included consistently more pores compared to the extruded portion from the compule tip, and in the final restoration (*p* < .001). The amount of porosity in the restorations differed amongst the tested materials, with the flowable bulk-fill composite showing the lowest amount of porosity (*p* < .01).

## 1. Introduction

Composite polymer resin (CPR) is today the most commonly used material for direct restorations in dentistry, with more than 260 million dental restorations placed annually [1]. The CPR for dental restorations is often contained in a compule for single-use, referred to as CPR compule in this study. The extensive use of CPR for dental restorations implies that small improvements to these materials can have vast implications concerning time and money saved for patients and clinicians. One of several strategies to improve the clinical performance of CPRs is to minimise the amount of porosity within the dental restoration [2].

A pore is a space-occupying area within or at the border of material and the amount of porosity describes the volume percentage of pores per volume unit (vol%). From a mechanical perspective, pores represent defects/flaws in material as it is a discontinued phase of the material with an e-modulus of zero [3]. There is no direct clinical evidence on how the amount of porosity may affect the clinical performance of CPR restorations. Still, ample data from *in vitro* studies indicate that restoration with embedded pores may be a clinical concern. An increase in the amount of porosity in the range of 1.5–3 vol% in a CPR reduces the compressive strength and compressive fatigue limit, estimated to be in the range of 30–50% [4], and the pores may be considered as critical defects associated with fracturing of specimens [5].

Moreover, the amount of porosity correlates with increased water sorption and consequently facilitates hygroscopic/hydrolytic activities such as swelling (separation of polymer chains), hydrolysis of constituents of the polymer chain, and degradation of the silane interface between filler and matrix in the CPR [6–8]. The effect of water on composite resin samples is a reduction in bi-axial flexure strength in water compared to freshly cured samples [8].

Large surface pores may contribute to secondary caries development if they are situated along critical regions of the restoration, but the size-threshold is debatable [9].

Estimates of the amount of porosity in CPRs varies between studies and falls mostly within the range of 0.5–4 vol% [10–13]. The inconsistency may be explained by material handling factors in the laboratory or clinic, as well as the inherent properties of the material itself as a function of qualities and quantities of oligomers and fillers. Additional factors that may introduce pores in the material are mixing and injection of composite resins into compules during the manufacturing processes, although the contribution of such factors remains unknown. Furthermore, the method used to identify pore diameters will likely influence the estimated amount of porosity [13,14]. One approach is based on sectioning samples of the dental restoration and compute the pore area in each section cut and extrapolate the counts to the full sample [13]. Such an approach is valid only if the pores are distributed homogenously in the sample and, unless the cut sections are extremely thin, one cannot establish the pore size distribution. Moreover, the technique is destructive and cannot be used to study pores in unpolymerised materials contained in, e.g. compules.

A non-destructive technique to identify pores in samples, which also enables estimating the pore size distribution, also known as differential pore volume distribution or porosity spectrum, is the use of x-ray microtomography, referred to as micro-CT in this article. The method is non-invasive, three-dimensional and can be used to appraise the amount of porosity both in polymerised and in unpolymerised specimens. While many studies have measured the amount of porosity in polymerised CPRs [10–13], nobody has – to the knowledge of the authors – undertaken studies of whether the unpolymerised material within CPR compules may include pores. Quantifying and characterising such pores can provide knowledge about potential sources of pores in dental restorations. With this background, the study objectives were to assess whether composite polymer resin delivered in compules include pores and the possible effect on the amount of porosity in dental restorations.

## 2. Material and methods

### 2.1. Composite polymer resins

Forty CPR compules were used, representing four different commercial products. Three products are marketed as a universal CPR: Herculite XRV Ultra (LOT # 5469627, Kerr, Bioggio, Switzerland), Ceram.X Universal (LOT # 1702000550, Dentsply Sirona, York, PA, USA), Tetric Evo Ceram (LOT# V47223, Ivoclar Vivadent, Schaan, Liechtenstein). One product is marketed as a flowable bulk-fill composite, SDR (LOT# 1701000793, Dentsply Sirona, York, PA, USA). All products were purchased from a commercial dental supplier in Norway (Dental Spar AS, Drammen, Norway).

### 2.2 X-ray microtomography

A high-resolution desktop micro-CT (Skyscan 1272, Bruker, Kontich, Belgium) was used for scanning the CPR compules (*n* = 40, 10 in each group). The scanning was performed with the isotropic pixel size of 12 µm for the CPR compules, the non-manipulated composite polymer resins and the dental restoration with the following settings: 100 μA, 100 kV voltage and a 0.11 mm Cu filtration. 360° rotation was used, with an angular step of 0.45°. At each step, a shadow projection 16-bit image was taken. The projection was an average of four images. Pixel binning was set to 3-by-3. Flat field correction was done before every scan.

The projection images were reconstructed into cross-sectional 8-bit bitmap file format images using NRecon computer software (Bruker, Kontich, Belgium). The same reconstruction settings (smoothing, beam hardening, histogram) were used for all materials within the same group. All analyses were conducted using proprietary software (CtAn computer software, Bruker, Kontich, Belgium).

### 2.3. Micro-CT analysis of compules

The scans of the CPR compules were segmented to establish the amount of porosity of the material contained in the horizontal top half and bottom half as well as in the central part (using the 2/3 of the total volume). The segmentation was done individually for each CPR compule and was defined from the first image slice of the compule not in contact with the extrusion lumen (top) or in contact with the bottom of the compule. Manual segmentation was required because the radiolucency of the CPR compule polymer was analogous to the radiolucency of air/pores, which made automized analyses challenging.

**Figure 1.**
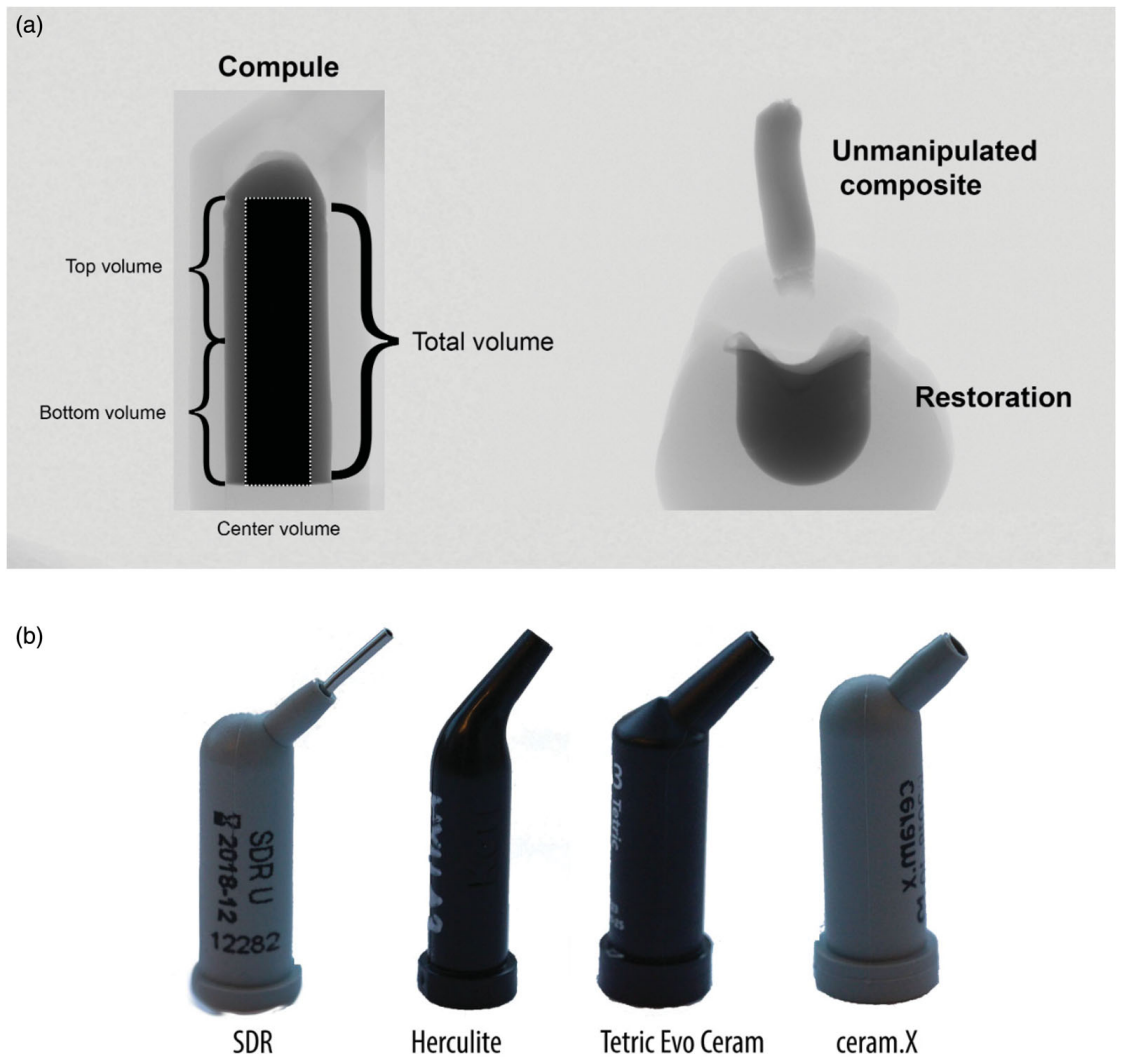
(a) Setup for the micro-CT analyses of the CPR compules (left), and the non-manipulated composite and the dental restoration (right) as seen in the micro-CT scanning software. The compule and the non-manipulated composite and dental restoration was scanned separately. The center volume include regions of the top and bottom volume. The extruded portion and the material placed in the typodonts were light-polymerised individually, but affixed together with wax before conducting the scans. (b) the CPR compules used in the study.

Threshold values of suitable greyscale for each group of CPR compules were defined by one investigator and subsequently agreed by a second investigator. The threshold values used were 184–255 for Tetric Evo Ceram, 171–255 for Ceram.X Universal, 110–192 for SDR and 155–219 for Herculite XRV Ultra. An overview of the scanning setup, the micro-CT analyses, and designs of the compules, is shown in Figure 1.

### 2.4. Cavity preparation in polymer teeth

Forty standardised cavities were created in upper first molar (#26) typodont teeth (AG-3, Frasaco GmbH, Tettnang, Germany) using a rigid tool setup on a milling table (FB-H, Demanders Verktygsfabrik AB, Virserum, Sweden). A Kestag High-Speed steel end mill with a diameter of 5 mm was mounted (Ibarmia B-35, Azkoitia, Spain) and a cylindrical cavity was prepared to a depth of 3.5 mm with a rounded cavity floor. A pointed bud, fine grit, diamond bur (Viking, Foss & co, Norway) was used to roughen the internal walls prior to bonding with Clearfil SE Bond (Kuraray, Osaka, Japan) according to the instruction for use.

### 2.5. Material handling

A licensed dentist with more than 10 years of clinical experience handled all materials and placed the dental restorations using the 40 CPR compules that had been scanned. All cavities were restored in teeth in the upper jaw model (AG3, Frasaco GmbH, Tettnang, Germany) mounted to a phantom head (P-6/3, Frasaco GmbH, Tettnang, Germany). Approximately 0.6 cm of the material was first extruded from the CPR compule and light-polymerised according to the manufacturer’s instruction by use of a calibrated light-curing unit (Bluephase style, Ivoclar Vivadent, Schaan, Liechtenstein). The cavities were next filled according to the manufacturer’s instruction for the respective materials. Instruments used during the manipulation of the composites were LM 4471-473 SI (1.5–2.5) ball burnisher and LM 482-702 SI, packer-modeller IC P (LM-Instruments, Pargas, Finland). The universal composites (Herculite XRV Ultra, Ceram.X Universal, Tetric Evo Ceram) were placed with three layers, and the flowable bulk-fill composite (SDR) was placed in one layer.

### 2.6. Micro-CT analyses of dental restorations

The volume of interest was segmented manually for each dental restoration because the radiolucency of the typodont tooth and the pores were too similar to automatize the analyses. A minimum of seven regions of interest (ROIs) was defined for each sample, with interpolation between images, to get an accurate volume of interest. The top/bottom slice to be analysed was determined in the same manner as for the compule analysis. The presence of pores was analysed using the same segmentation as for the compule, i.e. the total morphology, the horizontal top half and bottom half, and a central part (using the 2/3 of the ROI size of the total volume. The same threshold values were used in the analyses of the unpolymerised material in the CPR compules.

### 2.7. Data availability

The raw-data (projections and reconstructed images) obtained during the micro-CT analyses are available upon request to the corresponding author.

### 2.8. Statistical analysis

Normality (Shapiro–Wilk) and equal variance (Brown–Forsythe) tests were performed on the data to assess parametric assumptions. A Kruskal–Wallis One Way Analysis of Variance on Ranks was used to compare the amount of porosity in the material located in the compules, including the separate measurements of the material contained in the bottom, centre and top segments of the compule. and to compare the total amount of porosity in the dental restorations (Student–Newman–Keuls posthoc tests were used). Mann–Whitney Rank Sum tests were used to compare the total amount of porosity in the top region of the CPR compules and the non-manipulated composite samples. All statistical analyses were performed with α = 0.05. Data handling, statistical analyses and graphs were made with Sigmaplot 14 (Systat Software Inc, San Jose, CA, USA).

## 3. Results

### 3.1. Presence of pores

**Figure 2.**
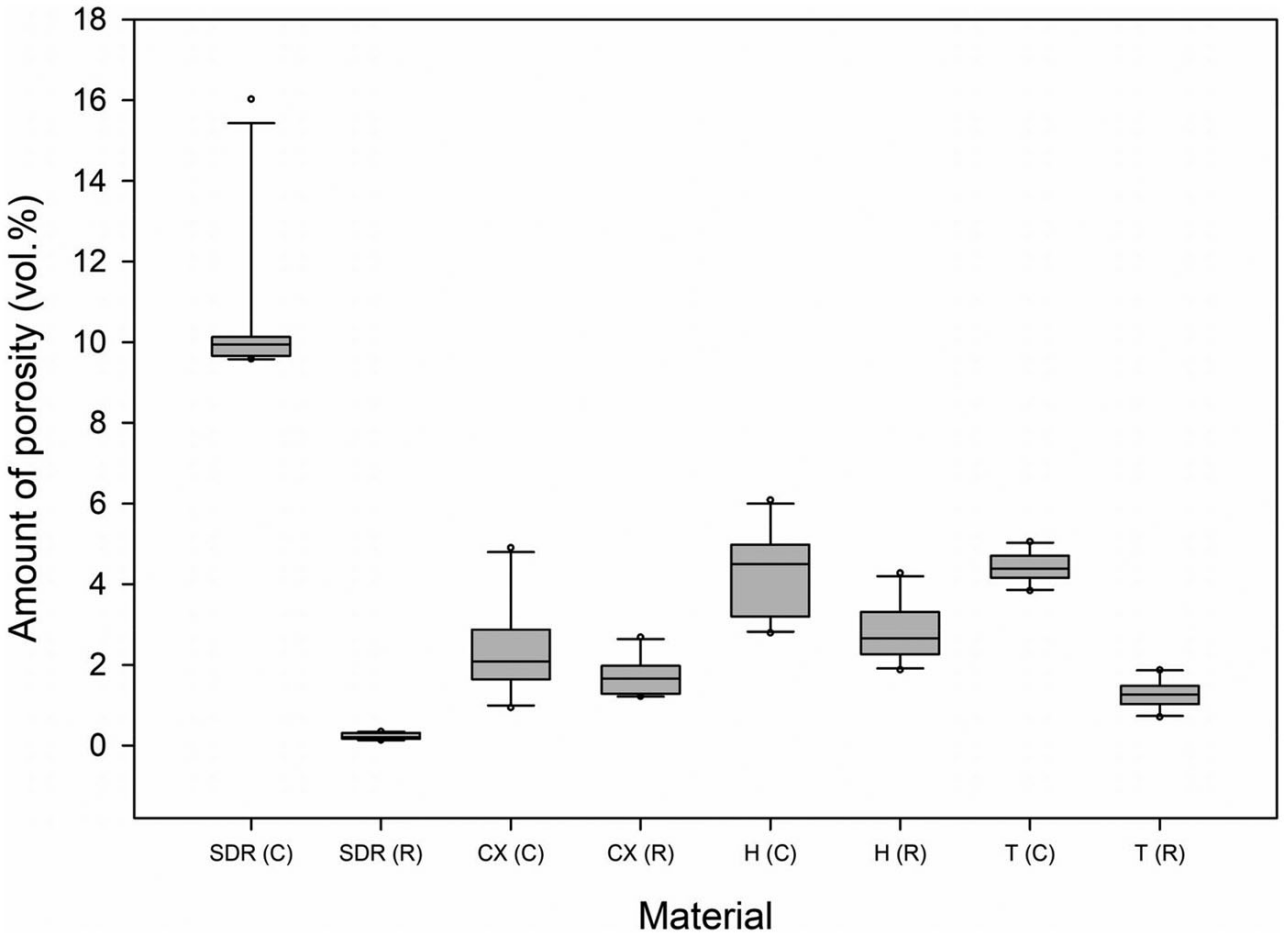
Boxplots of the amount of porosity (vol%) in the unpolymerised material in the compules (C) and in the dental restorations (R). Data shown are the average of 10 samples. Materials: SDR, Ceram.X Universal (CX), Herculite XRV Ultra (H), Tetric Evo Ceram (T).

Pores were identified in all compules. The amount of porosity (vol%) in the three universal composites differed from the flowable bulk-fill material (*p* < .001) (Figure 2). Within each material group, the amount of porosity varied among individual samples, respectively within ranges of approximately 10–16 vol% (flowable bulk-fill) and approximately 0.1–6 vol% (universal composites). The amount of porosity at the interface (i.e. porosity in contact with the compule or typodont inner walls) was higher than the amount of porosity totally embedded in the material (Table 1).

**Table 1.** Amount of porosity (vol%) in the unpolymerised material contained in the compule and the polymerized material in the dental restorations (averages of *n* = 10).

	Peripheral part porosity (vol%)		Closed porosity (vol%)		Sum amount of porosity (vol%)	
Compule	Average	Standard deviation	Average	Standard deviation	Average	Standard deviation
Ceram.X Universal	2.05	0.69	0.32	0.82	2.37	1.19
Herculite XRV Ultra	3.94	0.91	0.40	0.40	4.34	1.03
SDR	10.49	1.95	∼0	∼0	10.49	1.95
Tetric Evo Ceram	4.39	0.37	0.03	0.01	4.42	0.37
Dental restoration						
Ceram.X Universal	1.59	0.34	0.14	0.24	1.73	0.48
Herculite XRV Ultra	2.29	0.47	0.54	0.39	2.83	0.72
SDR	0.21	0.06	0.02	0.04	0.23	0.08
Tetric Evo Ceram	1.19	0.32	0.08	0.06	1.27	0.35

The amount of porosity in contact with the inner walls of the compule and the cavity walls in the typodont is labelled as ‘peripheral part porosity’, while the amount of porosity totally embedded in the material/restoration is labelled ‘closed porosity’.

There was a statistically significant difference between the amount of porosity of the dental restorations made from the different materials (*p* = .019 for Ceram.X Universal versus Tetric Evo Ceram and *p* ≤ .001 between all other comparisons). The least amount of porosity was seen in the restoration made from the flowable bulk-fill composite (SDR), while the dental restorations made with Herculite XRV Ultra had the largest variance of the amount of porosity in the compules (Figure 2).

### 3.2. Location of pores in the compule

**Figure 3.**
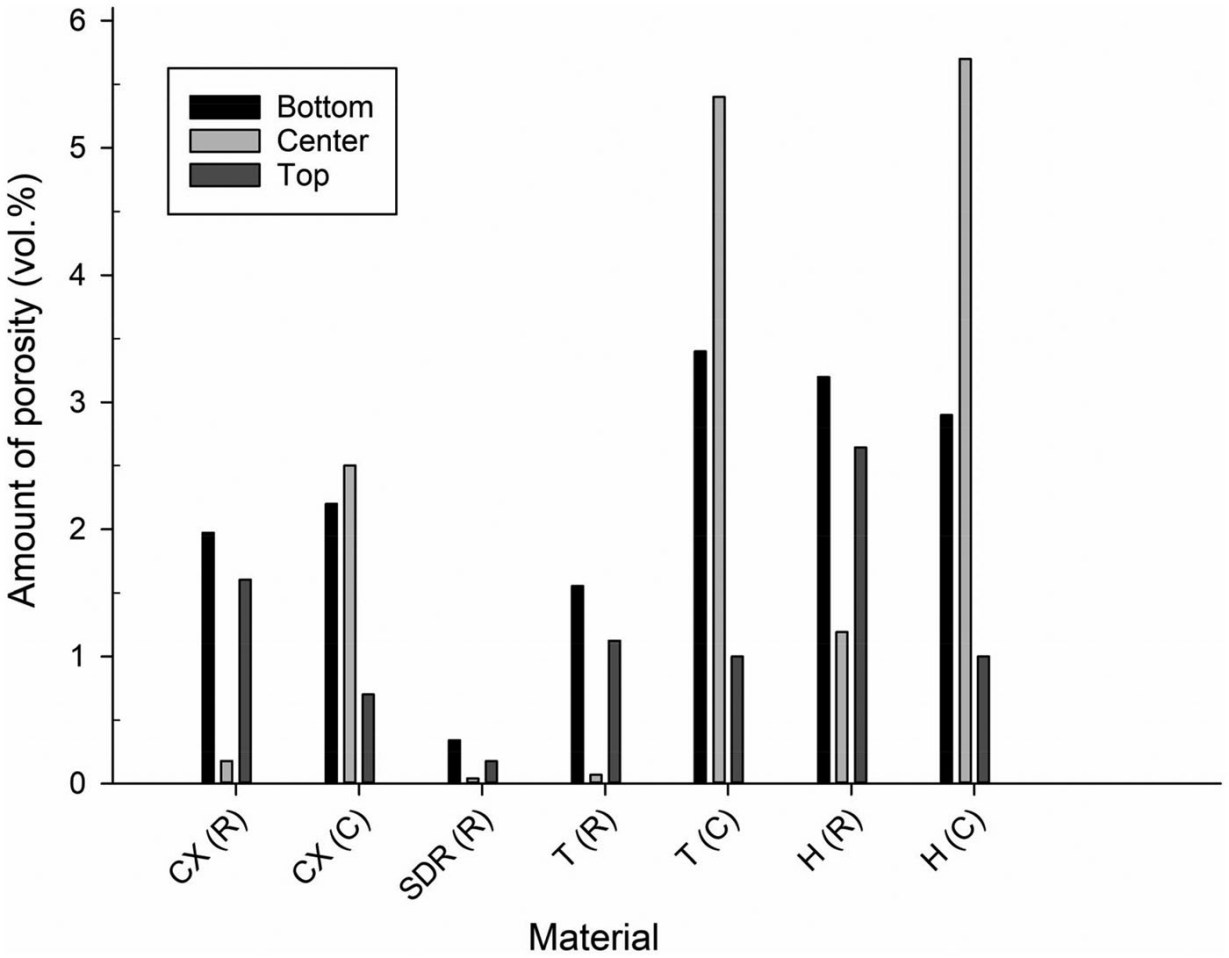
The amount of porosity (vol%) in the unpolymerised material in the top, centre and bottom parts of the compule (C) and in the polymerized material in the top, centre and bottom of the dental restorations (R). Data shown are the average of 10 dental restorations. Materials: SDR, Ceram.X Universal (CX), Herculite XRV Ultra (H), Tetric Evo Ceram (T). Data for SDR (C) omitted from the figure. Results for SDR (C): bottom: 20.4%; top: 0.3%; center: 20.1%.

The pores were not homogeneously distributed in the unpolymerised material in any of the CPR compules (Figure 3). In the universal composite resin compules (Herculite XRV Ultra, Tetric Evo Ceram, Ceram.X Universal), most of the pores were clustered along the inner walls with few pores in the centre part. In contrast, the flowable bulk-fill composite resin (SDR) compules showed pores situated in the bottom and centre 2/3 of the compule.

### 3.3. Location of pores in the dental restoration

The location of pores within the dental restorations was not homogenously distributed for any of the materials (Figure 3). Few pores were identified in the centre bulk of the dental restorations, while they predominated in the zones intimately in contact with the cavity bottom and cavity walls.

### 3.4. Amount of porosity in the compule versus in the extruded portion and in the restoration

**Figure 4.**
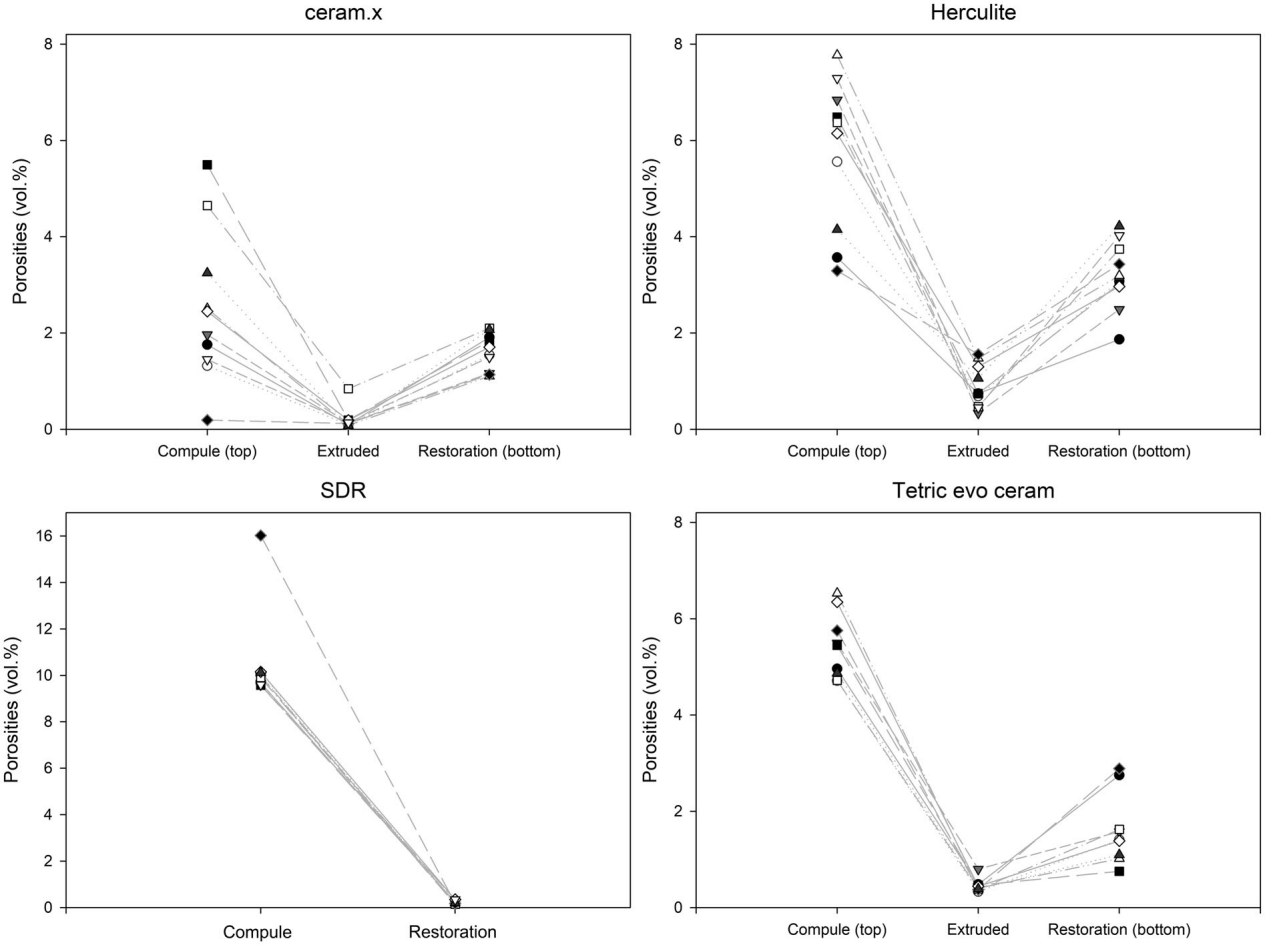
The relationship between the amount of porosity (vol%) in the unpolymerised material in the compule, the non-manipulated material and the dental restoration on a sample level (*n* = 10). The lines between data points are only shown for clarity and do not represent an assumed function between variables. Note that the upper limit of the Y-axis of flowable bulk-fill composite (SDR) is higher than the Y-axis of the universal composites (which are similar). There is no linear relationship between the amount of porosity in the compule and the amount of porosity in the dental restoration.

The amount of porosity in the top part of the CPR compules and in the extruded portion differed (*p* ≤ .001 for all samples). The average amount of porosity in the extruded portion was 0.2%, 0.5% and 0.8% for Ceram.X Universal, Tetric Evo Ceram and Herculite XRV Ultra, respectively. The amount of porosity in the unpolymerised material in the compules versus in the extruded portion versus in the dental restoration did not correlate linearly (Figure 4).

### 3.5. Pore size distribution and accumulated surface area of all pores

**Figure 5.**
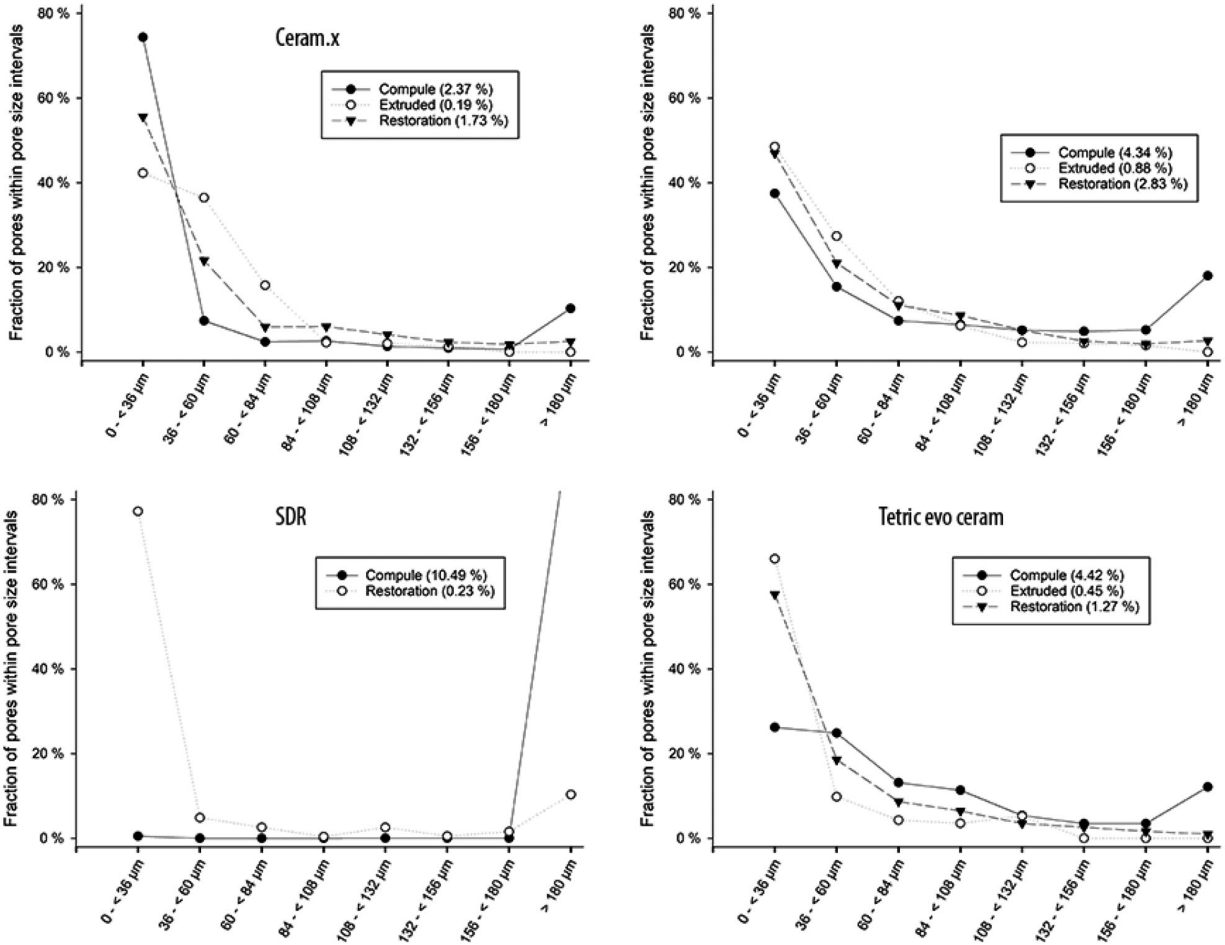
The pore size distributions in the unpolymerised material in the compules and in the polymerized material, respectively in the extruded portion from the compule tip and in the material used to restore the cavity in the typodont. The Y-axis is the percentage of pores within the stated range in micrometres (µm) described along the X-axis. The lines between data points are only shown for clarity and do not represent an assumed function between variables. Data shown are the average of 10 samples.

The pore size distribution varied among the investigated materials (Figure 5). The Ceram.X Universal compules included a more substantial proportion of pores in the < 100 µm range. However, some Ceram.X Universal compules included additional large diameter pores (Figure 5). Similar outliers were also identified amongst the other universal composite materials. All the flowable bulk-fill composite (SDR) compules had only one or two large diameter pores, presumed to be air bubbles.

In the extruded portion, most pores were <100 µm for all the materials. The extruded portion made from the outlier compules shown in Figure 5, did not contain a higher number of large diameter pores than the average. However, in another sample, more than ¾ of all the pores in the extruded portion had a larger diameter than the average.

**Figure 6.**
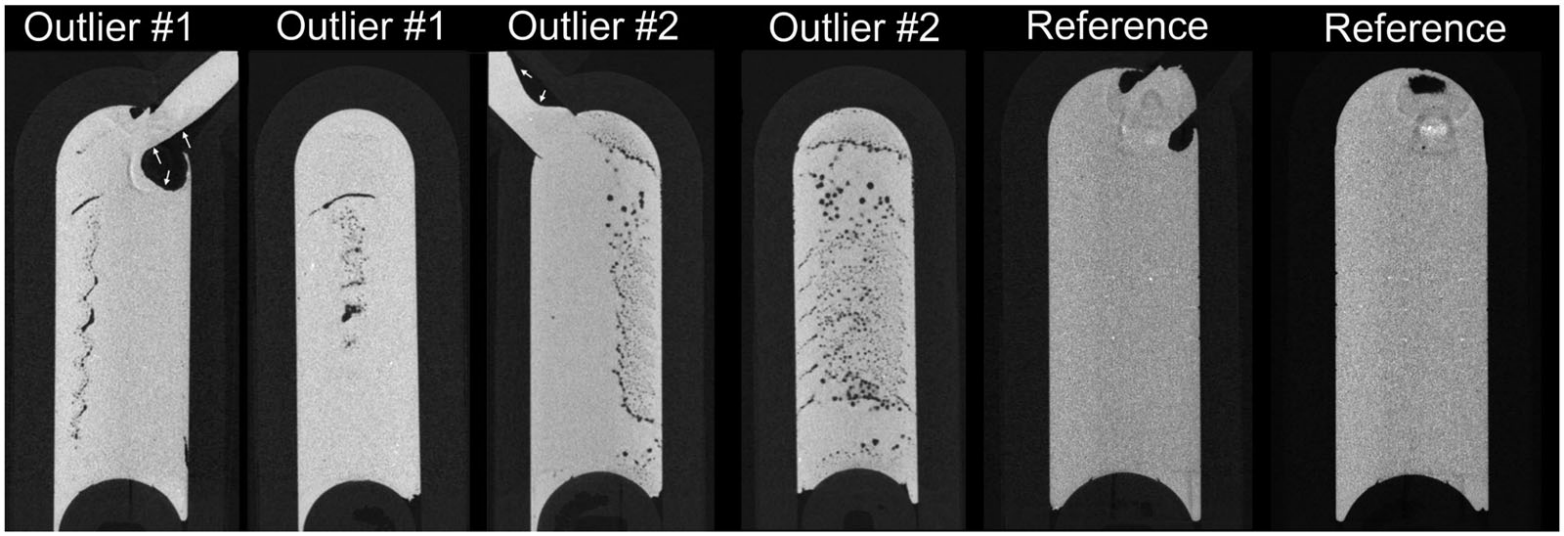
Examples of two outlier compules in the Ceram.X Universal group viewed in Dataviewer software (Bruker, Kontich, Belgium) from the coronal and sagittal view. Note the large pore/air bubble in the extrusion lumen of the compules (white arrow showing the border between material and lumen). Right images show a third compule of Ceram.X Universal with less than the average amount of porosity.

Concerning the dental restorations, most of the pores were <100 µm in diameter, for all the investigated materials. However, there were some outliners in all of the groups. For example, in the flowable bulk-fill composite (SDR) group, one sample included pores, of which 60% were within the 348–372 µm range. In the dental restorations made from sample #6 from Ceram.X Universal (Figure 6), more than 3/5 of all pores had a diameter larger than in the average sample.

**Figure 7.**
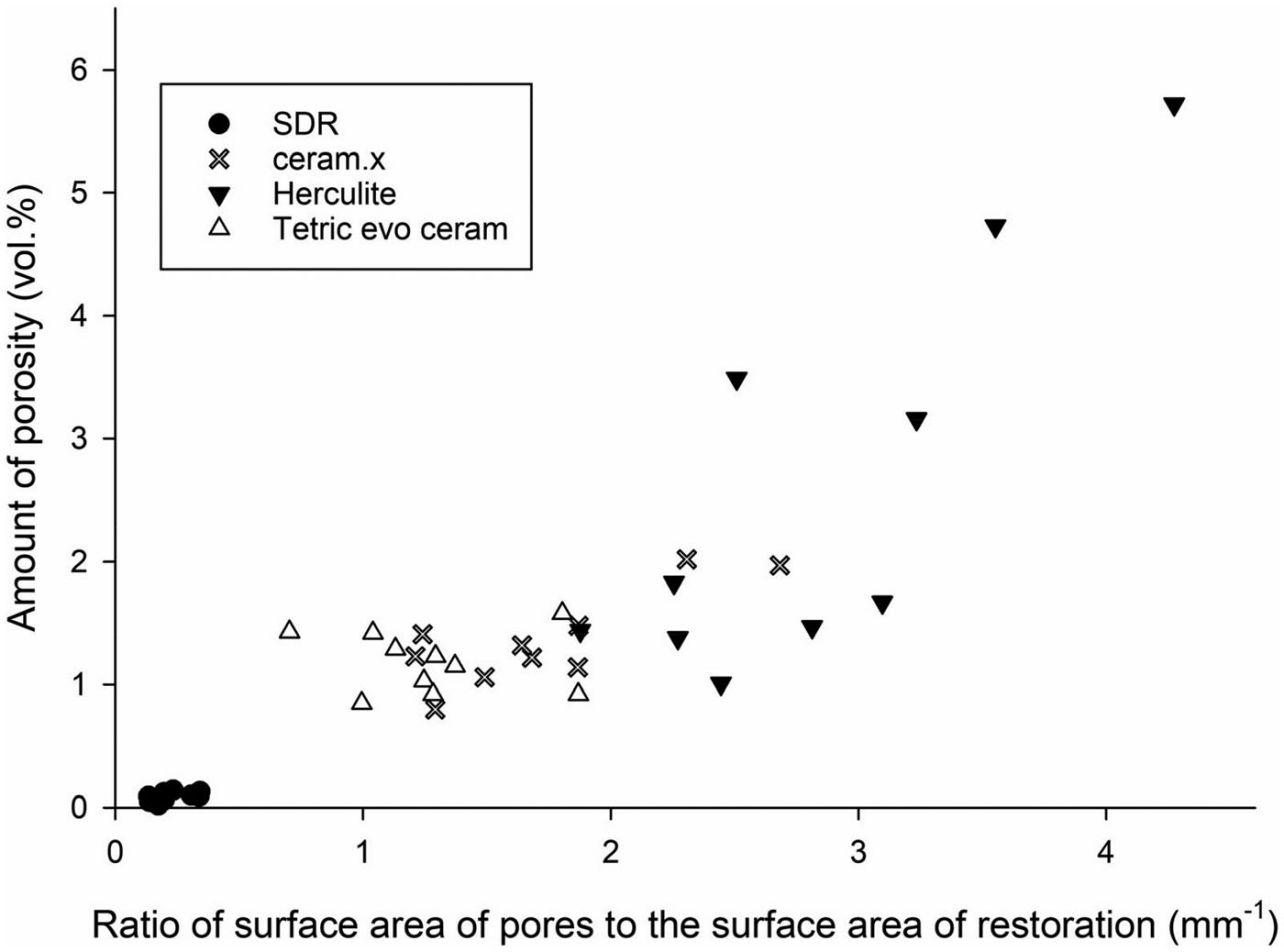
The relationship between the total amount of porosity (vol%) in restorations made with the four investigated materials and the accumulated surface area of all pores adjusted for the total volume of restoration analysed (*n* = 10 for all groups).

The accumulated surface area of all pores, adjusted to the total volume of the dental restoration, were in average 1.36 mm^−1^ (Ceram.X Universal), 2.59 mm^−1^ (Herculite XRV Ultra), 0.09 mm^−1^ (SDR) and 1.18 mm^−1^ (Tetric Evo Ceram). The surface area of all pores is a function of both the amount of porosity (vol%) and the pore size distribution (Figure 7).

## 4. Discussion

Creating CPR restorations without pores or voids is challenging, independent of application technique and material [15]. In the present study, we investigated if the composite resin in CPr compules included pores and if these influenced the amount of porosity in restorations. All the CPR compules included pores, but there was no direct linear correlation with the amount of porosity in the final restoration.

The pores in the CPR compules were not distributed homogeneously. In the universal composites, the pores were mainly situated towards the periphery, while in the flowable bulk-fill material, the pores were primarily located towards the bottom. This finding indicates that averaging the amount of porosity on cross-sectional photographs should be done with caution since the majority of pores are situated along the interface zone between the material and the compule walls for all the investigated materials. Pores in the peripheral area of the universal CPR compules may be associated with the low surface energy of polyvinyl chloride, polyethene, polypropylene and other polymers used in CPR compules, as low surface free energy is related to poor surface wetting. Yet, no studies on the surface energy of CPR compules have, to the knowledge of the authors, been published. Furthermore, the chemistry of the composite material may also influence the wetting of the compules as different materials have varying filler loading and monomers that affect hydrophobicity and viscosity [16].

There was a significant difference between the amount of porosity in the CPR compules of the universal composites and the flowable bulk-fill, but not between the individual universal composites. The pores within the flowable bulk-fill (SDR) seem to be one or several air bubbles because they changed shape and moved between repeated scans of the same sample. With regards to the universal composites, the presence of outliers, as shown in Figure 6, suggest that the amount of porosity in the unpolymerised material in compules varies within the same batch. This highlights that there is room for improvements in the manufacturing process of CPR compules.

The amount of porosity in the unpolymerised material in the compule differed from that in the extruded portion and in the dental restoration for all the investigated materials. The relative lower amount of porosity identified in the extruded portion, in comparison with the higher amount of porosity found both in the unpolymerised material in the compule and in the polymerised material in the typodont, suggest that the amount of porosity in the compule do not bear a linear relationship with the amount of porosity in the dental restoration (Figure 4).

The location of pores and the pore size distribution and the amount of porosity in the dental restoration varied significantly amongst the four products. The low-viscous flowable bulk-fill composite (SDR) had fewer pores, and much less variation in the amount of porosity between samples, compared to the high-viscous universal composites (Figure 2). These findings are in line with results by Lagouvardos *et al.* (2015) which concluded that high-viscous materials, such as universal composites, have a higher the amount of porosity compared to low-viscous materials [17]. Yet, others have found the opposite, i.e. that high-viscous materials produce less amount of porosity than low-viscous materials [12,14]. Regarding the location of pores in the restoration, an increased amount of porosity could be visually identified between increments when using universal composites. The association between amount of porosity as a function of incremental layering of material has been shown previously [18,19].

The amount of porosity detected in the dental restorations made from universal composites is in alignment with other publications. In the present study, we used bonded, standardised, rounded, cylindrical-shaped cavities that were filled under simulated, clinical conditions in phantom heads. However, many studies on the amount of porosity in CPRs are conducted under less clinical conditions. For example, in a micro-CT based study on Grandio and Filtek P60 universal composite dental restorations, the amount of porosity detected (i.e. 0.05–0.09%) was much lower than in our sample [14]. In the particular study, the dental restorations were light-polymerised under pressure (with clamps) in Teflon moulds, which may explain the low amount of porosity detected.

The possible impact of the presence of pores in critical areas of the tooth restoration interface is uncertain. Maske *et al.* concluded that a threshold gap size for risk of secondary caries is less than 30 µm [9]. Even though the majority of pores in the restorations in the current study were situated in proximity to the tooth/restoration interface (Table 1), approximately ½ to ¾ of the pore sizes were within the 0–36 µm diameter range. The actual range is likely closer to the 24–36 µm diameter, as the isotropic pixel size of 12 µm used in our study do not yield a spatiel resolution of 12 µm due to factors such as the partial volume effect and noise [20]. Yet, whether pores in this diameter range may have a potential to contribute to caries development if they are situated in a critical region of the restoration remains to be demonstrated.

In our sample, the surface area of all pores within the examined restorations was quite large since the pore sizes were quite small. Yet, despite a similar amount of porosity, the accumulated surface area of all pores can vary between samples due to the differences in pore size distribution (Figure 7). An approximation of the total volume, as well as the accumulated surface area of all pores under the assumption that these are spherical, is $\sum \frac{4\pi r^{3}}{3}$ and $\sum 4\pi r^{2}$ respectively.

A larger accumulated surface area of all pores implies a more substantial potential for hygroscopic and hydrolytic effects. The accumulated surface area of all pores in the universal composites was more than 10 times larger than the accumulated surface area of all pores in the flowable bulk-fill composite (SDR), mainly due to higher amount of porosity, suggesting that the universal composites may be more susceptible to internal hygroscopic/hydrolytic effects. However, given that resolution of the device used in the current study was 12 µm per voxel, there is a high likelihood that an unknown amount of porosity consisting of pores below this diameter remains undetected. Water penetrates polymer networks on a submicron scale independently of the diameter of pores [21]. Water sorption and solubility test of CPRs (both universal and bulk-fill) show that these variables are highly influenced by filler loading and hydrophilicity of the resin matrix [16]. Yet, the differences in sorption can also be explained by different amounts of porosity. Further studies are needed to clarify possible effects of amount of porosity and pore size distribution on permeability and material degradation.

In conclusion, pores are heterogeneously present in the unpolymerised material in CPR compules. We did not detect a linear relationship between the amount of porosity and pore size distribution for pores ≥12 µm in the compule and the dental restoration. For the restorations made with universal composites, the low amount of porosity seen in the non-manipulated CPR, combined with the variability in the amount of porosity in the final restoration suggests that the manipulation and handling of these materials generate pores and voids in the material. In contrast, the low variability in the amount of porosity observed in the restorations made with the flowable bulk-fill composite (SDR) may be an indicator that the materials are less technique-sensitive.
